# Fostering Reflexivity in Medical Students: Is Patient Engagement a Promising Avenue? A Qualitative Case Study

**DOI:** 10.1177/23821205251324295

**Published:** 2025-03-21

**Authors:** Julie Massé, Sarah Numainville, Marie-Claude Tremblay

**Affiliations:** 1 12369 Faculty of Medicine, Université Laval, Quebec City, QC, Canada; 2 Faculty of Nursing, Université Laval, Quebec City, QC, Canada; 3 607356 Vitam, Centre de recherche en santé durable, Quebec City, QC, Canada

**Keywords:** reflexivity, medical education, teaching and learning, group learning, patient engagement, patient participation, interventional research, qualitative research

## Abstract

**Background:**

Reflexivity enables individuals to analyze a situation based on past experience to develop other ways of thinking and perspectives for action. Reflexivity is therefore crucial for the improvement of professional practice. In medical education, recent studies have identified patient engagement as a promising strategy for fostering reflexivity in students; however, few evaluative studies have explored such a link. This article describes the reflexive effects of an intervention that engages patients in small-group discussion workshops about ethical, moral, and social issues arising from practice (as part of an undergraduate medical course at Université Laval) and presents the main processes involved in producing these effects.

**Methods:**

The study subscribes to a qualitative case study design. Cases are three groups that received the intervention in winter 2021. Data collection involved semi-structured interviews and non-participatory observation. Analysis entailed within-case and cross-case analysis. The study mobilizes Sandars' proposition of a three-stage reflexive process which is enhanced with other models of reflexivity.

**Results:**

The main reflexive effects and processes involved: (i) better understanding disembodied theoretical content, (ii) awareness of the limits of the clinical view for grasping complex situations, (iii) questioning one's convictions about the self and the profession, and (iv) awareness of the patient-doctor social distance. When considering concrete implications for action, reflexive effects refer to a patient-centered approach, implying other ways of doing, being, and thinking as a physician.

**Conclusions:**

This study was an opportunity to identify patient engagement in discussion workshops as a promising avenue to foster medical students' reflexivity and to better understand its whys and hows. It sheds new light on patient engagement's relevance and value in medical education. By identifying factors influencing the reflexive process, it also provides concrete support to medical schools wishing to commit to transformative educational postures and approaches involving patients.

## Background

Conceptually derived from social sciences and then mobilized in several disciplinary fields, reflexivity is generally understood as “an intentional intellectual activity in which individuals explore or examine a situation, an issue or a particular object on the basis of their past experiences to develop new understandings that will ultimately influence their actions.”^1^^(p539)^ Since it is conducive to the deployment of alternative ways of thinking and new perspectives on action, reflexivity is especially crucial to improving the training of healthcare professionals and to the subsequent deployment of professional practice in tune with collective needs and expectations. Mann et al^2^^(pp595-596)^ justify in these terms the growing interest in the concept of reflexivity in health professions education:

Today's health care professionals must function in complex and changing health care systems, continuously refresh and update their knowledge and skills, and frame and solve complex patient and healthcare problems. Preparing professionals who possess these capabilities is correspondingly complex. Reflection, [reflexivity] and reflective practice are frequently noted in the general education literature and are increasingly described as essential attributes of competent health care professionals who are prepared to address these challenges.

Despite recognition of the fundamental importance of reflexivity, medical schools still appear ill-equipped to support its proper unfolding among medical students.^2–5^ In particular, studies highlight the persistent lack of evidence needed to empirically inform the development of reflective pedagogical interventions and innovations.^2^ They also point to the deleterious effects of the lack of theoretical and conceptual consensus regarding reflexivity, which leads to uncertainty as to the best ways to support its development in medical learners.^3^ Others outline how the biomedical and competency-oriented mindset—which is still dominant in most medical schools—tends to promote utilitarian and self-centered conceptions and pedagogies of reflexivity, which are fundamentally at odds with the epistemology underlying reflexivity theories focusing primarily on experience as the reflexive object and on the social and situated dimensions of reflexivity.^4,5^

Meanwhile, recent decades have seen a significant transformation in approaches to care in line with a renewed vision of relationships between patients and the healthcare system often referred to as *patient engagement.*^6,7^ It is the culmination of a logical evolution, over several decades, of ways of thinking about health and organizing health care and services.^8,9^ This renewed vision is rooted in the democratic and emancipatory ideals of participation. It recognizes the importance and relevance of patients' specific knowledge and expertise^10^ as well as patients' right and legitimacy to teach about their realities and to co-define health education priorities.^11^ Conceived in these terms, *patient engagement* has conceptual similarities with the neighboring idea of *patient involvement*. Indeed, the two concepts are often used interchangeably, contributing to the “story of terminological fuzziness” that emerges from the scientific literature on the subject.^12^^(p2685)^ Like other authors,^13^ we propose a certain distinction between these two. For us, patient involvement represents the set of concrete actions taken by patients in different health contexts, where they are invited to take on active roles. These actions are the result of a broader multi-stakeholder process of patient engagement at the interface between the system (where structures and actors are being called on to enable and support active patient roles) and the patients (embracing such roles).

Even if patient engagement in health training and medical education appears to be a rapidly growing field, it still has yet to be fully studied and understood.^14–17^ Many studies in the field focus on the nature of the learning gained through patient engagement and its quantitative measurement, thus limiting our in-depth understanding of the potential benefits of such engagement, the mechanisms involved and the limits of this pedagogical approach. Furthermore, Usher and Denis^12^^(p2688)^ highlight how the actual body of literature on engagement “reduces the story's credibility by dispensing with context” since very few studies characterize and study the influence of the broad learning context on student learning with patients.

In this study, our primary premise is that patient engagement could be a promising pedagogical avenue for overcoming the challenges experienced in medical schools and adequately supporting the development of reflexivity among medical learners. This article thus aims to present the results of an interventional research study interested in (i) the perceived reflexive effects of a transformative, situated, and empirically and theoretically grounded educational intervention engaging patients in small-group discussion workshops as part of an undergraduate medical course at Université Laval (UL), Québec, Canada, and (ii) the main processes involved in producing these effects.

Our premise linking patient engagement to reflexivity has both strong empirical^14^ and theoretical relevance since reflexive learning is expected to occur through experience in context, in relation with others,^4,5^ and presupposes a process of interactive and sensible sharing of knowledge and perspectives.^18–21^ However, very few studies specifically explore patient engagement's reflexive effects in the medical education context. We thus consider that medical education literature will benefit from such a rigorous evaluation study to help bridge the knowledge gap while deepening our contextualized understanding of the learning processes linking patient engagement in teaching and the development of reflexivity in future physicians. This will help medical schools optimize this practice and, ultimately, make it an effective means of transforming healthcare education and practices.

## Methods

### The patient engagement educational intervention and its implementation context

This study is part of a larger project aimed at developing, implementing, and evaluating an intervention involving the engagement of patient-teachers in an undergraduate medical course at UL, which is described and documented elsewhere.^14,22–24^ At UL, the medical program begins with an undergraduate doctorate (4 or 5 years), which is the first step on the academic path to becoming a physician. It is based on a competency-based approach that aims to enable future doctors to acquire the fundamental skills linked to the CanMEDS roles: Medical Expert, Communicator, Collaborator, Leader, Health Advocate, Scholar and Professional.^25^

UL Faculty of Medicine's strategic documents mention reflexivity as an essential approach to address contemporary social issues and develop key skills promoted by the CanMEDS roles.^26,27^ That said, notwithstanding the explicit acknowledgment of the importance and relevance of reflexivity and reflexive practice, teaching methods promoting reflexivity are still limited to self-centered strategies and self-assessment exercises aiming to make students aware of their learning trajectory and their professional identity. In response to this shortcoming, we came up with an innovative way of fostering reflexivity in students through a patient engagement intervention. The course *Médecin, Médecine et Société* II (MED-1210), was targeted for the implementation of the intervention.

The course MED-1210 is part of a sequence of four compulsory courses called “Médecin, Médecine et Société” (free translation: Physician, Medicine and Society) designed to familiarize future physicians with various ethical, legal, moral and social aspects of their future medical practice. It is offered to more than 200 medical students each Winter session (January-April). The undergraduate students enrolled in the course MED-1210 are in their first year of the medical curriculum. At this stage, students are learning the very basics of the profession and have had few opportunities to think more critically about their future medical practice. With a focus on the doctor-patient relationship, the MED-1210 course's main objectives are thus specifically (i) to develop student knowledge, skills and attitudes related to professionalism and communication and (ii) to engage students in critical thinking about their future practice. It thus distinguishes itself from most courses in the undergraduate curriculum that focus on the biomedical aspects of practice.

The course uses a variety of teaching methods (lectures, online courses, and workshops). Workshops were more specifically targeted by the intervention. They consist of a series of five 2-h small group discussion sessions involving 10-12 students. During those sessions, students are asked to collectively deliberate on legal, ethical, moral and social issues that may arise in the context of their medical practice based on fictitious scenarios presenting clinical cases developed by the course leaders. Table 1 summarizes the workshop themes and activities.

**Table 1.** MED-1210 workshop themes and activities.

	THEMES AND ACTIVITIES
Workshop 1	Deliberative discussions: Patient-centered clinical method
Workshop 2	Deliberative discussions: Beneficence and non-maleficence
Workshop 3	Deliberative discussions: Professional secrecy and confidentiality
Workshop 4	Deliberative discussions: Free and informed consent
Workshop 5	Summative integration activity: Student teams' presentation of their deliberative analysis of a clinical case followed by a question session and a group discussion

Those workshops are moderated by physician-instructors (practicing physicians or residents) whose formal mandate is to host the workshops; facilitate discussions between the participants; teach formal content related to the legal, ethical, and deontological aspects of medical practice; and assess students' learning. As part of the intervention, patients were invited to actively participate in the deliberative discussions by representing alternative perspectives rooted in their specific knowledge and experiences with illness and the healthcare system, to confront their own ideas and those of others and to ask questions that might prompt reflexivity in students. It was thus *a priori* stated that, within the intervention, the physician-instructors were to remain solely responsible for the design and the running of the workshops and that the role of patients would be that of participants in the discussions. At the same time, in a context marked by difficulties in recruiting instructors and maintaining their participation over time, course leaders were concerned about preventing the intervention from excessively adding to the instructors' workloads.

The course MED-1210, in the version that received the intervention, was marked by amendments made to the curriculum in the context of the COVID-19 pandemic and the associated public health requirements. This context has led to the cancellation of the clinical observation traineeships that normally take place prior to the course. For many students, the intervention thus offered a very first contact with real patients within their medical curriculum. Also, given the restriction of face-to-face contacts, the curriculum for most students was delivered exclusively online, usually via large group sessions, with an impact on the bonds the students forged with each other and on their sense of belonging to their cohort and program.

The intervention was developed and implemented based on the results of a literature review^14^ and the work of a steering committee involving patients (*n* = 4), students (*n* = 2), a course leader, and members of the research team (*n* = 3). The committee's mandate was to co-define the main practical modalities of the intervention considering the particularities of the local context and the main concerns of those involved. As a result of this work, 14 patient-teachers participated during the Winter semester of 2021. Recruitment was carried out using a variety of strategies drawing on the formal and informal networks of the patients involved in the steering committee. The characteristics sought in patient-teachers included: (i) significant experience (past or recent) in using healthcare, either as a patient or as a caregiver; (ii) an interest in supporting the training of future doctors; (iii) an ability to step back from experience, to question oneself and to allow oneself to be questioned in relation to that experience; and (iv) good communication skills coupled with the ability to speak in small groups. Given the nature of the intervention, which required patients to read, understand and reflect on clinical cases, a certain level of literacy was also considered for selection. Team members met with all those who expressed an interest to assess their fit with these criteria, to ensure they fully understood the nature of the commitment required and to give them the opportunity to ask questions. In the end, the patients recruited had varied profiles in terms of gender, age and lived experience (people living with a chronic illness, disability, mental health disorder, people in situations of social exclusion or poverty, etc), which corresponded to the steering committee's desire to allow students to meet with a range of patient realities and contexts.

Several strategies were implemented to support patient engagement throughout the intervention. For instance, a non-mandatory welcome event was organized to allow everyone to get to know each other and for patients to confirm their interest in participating. In addition, a compulsory three-hour orientation session was developed and offered to patients prior to the workshop sequence. This session was an opportunity to inform patients about the medical training curriculum at UL, the MED-1210 course, the research project and the patients' expected role within the workshops. They were given tools to support active and reflective participation and, as requested by the patients from the steering committee, had the opportunity to practice upstream in a safe environment. In the context of the COVID-19 pandemic, online workshops also meant addressing technological content with patients, notably concerning the use of videoconferencing platforms. The day before each workshop, non-compulsory peer preparation sessions were scheduled. Facilitated by the first author, these sessions were an opportunity for patients to meet up to read clinical cases together, collectivize their experiences and initiate a group reflection on what these cases evoked from their specific points of view, which they could then draw on during the upcoming workshop.

During the workshops, patients were placed in groups of two to facilitate participation. Physician-instructors were informed in advance of patient participation in workshops. Only those instructors who gave their consent welcomed patients into their workshop groups to promote a positive experience for all. Limited time was allotted to the research team to convey information to the physician-instructors about the intervention and its potential implications for their teaching during the instructors' orientation meeting planned by the course leaders.

All patient-teachers received financial compensation for their participation within the intervention. An online closing event was also organized. During this event, faculty governance members, students and physician-instructors were invited to express their gratitude to patients for their participation. All patients received certificates attesting to their participation as patient-teachers.

### The study design

This study subscribes to a qualitative instrumental case study design based on Stake's proposed methodology.^28,29^ The choice of such a qualitative design is consistent with the conclusions drawn from the quantitative component of our larger study,^22^ which was based on pre- and post-intervention surveys with a control group (using a translated and adapted version of the *Reflexive Practice Questionnaire*^30^). Consistent with most medical competency statements, this quantitative component was based on the premise that reflexivity is a generic transversal competency that can be decontextualized from lived experience and measured by a standardized instrument that breaks reflexivity down into fragmented behaviors; however, this idea is increasingly challenged by pedagogues, who tend to see it as a reductionist conception of reflexivity, which should rather be understood as intrinsically linked to the epistemology of the knowledge concerned and to the sensitive experience.^4,31^ Consistently, we have come to propose a qualitative approach that might be more relevant for finely documenting qualitative changes in thinking in all their subtlety and complexity.^2,4^

### Researcher's reflexivity

The first author (JM), as the leader of the intervention and research process, approached the project with a marked concern for issues related to epistemic injustices.^32^ She thus adopted a critical stance regarding traditional social dynamics in medical education rooted in the culture of a healthcare system that was fundamentally built based on scientific knowledge.^8,33,34^

This approach has translated into an awareness of her position of power vis-à-vis patients as a young, academic, white, educated woman with a favorable health status and a commitment to put in place research and intervention practices conducive to the fair recognition of patient knowledge. She therefore anchored her interactions with patients in a posture of humility, openness and sensitivity, recognizing the value, relevance and also the highly emotional nature of people's experiences of illness and the healthcare system. A great deal of attention was therefore paid to developing an authentic and committed relationship with patients.

JM was also aware of her position of power in relation to the students. Through her interactions with students, she was careful to adopt a posture marked by openness, listening and humility. She presented herself as a peer-student eager to engage in a discussion about the learning experience. She also sought to reassure students that the interview was a safe place to look critically at training, given the confidentiality of individual data and the independence of the research process.

### Theoretical and conceptual background: conceptualizing reflexivity

The theoretical and conceptual foundations of this study are broadly based on the iterative and recursive three-stage reflexive process proposed by Sandars^18^ enhanced by the broad assumption made by other authors^1,4,35–41^ of different levels of reflexivity: a formative level and a critical level, as named by Tremblay et al.^1^ *Stage 1 (Noticing)* corresponds to the initial awareness, where the individual recognizes that their mental models and theories of action are being challenged by a particular situation or experience. *Stage 2 (Processing)* refers to the individual's initiation of a process of meaning-making about self and the experienced situation. It refers to the individual's search for the reasons why the experience does not fit one's expectations. *Stage 3: (Consideration of implications for action)* enables the individual to reinvest one's reflection in practice by modifying one's action. At the formative level, such a reflexive process would enable healthcare professionals to cope with the complexity of their professional practice by supporting the production of new action-based technical and professional knowledge to support the further improvement of professional practice.^42–44^ That said, such formative reflexivity enables the improvement of professional practice from a perspective that omits the social and political forces at play within the complex health ecosystem.^1,4^ Critical reflexivity is thus aimed at “raising the professional's awareness and critical conscience from a broad social system perspective.”^1^^(p540)^ Operating mainly from the perspective of critical theory,^1^ such reflexivity would imply the questioning of one's premises of action and, more broadly, of the entire field of practice. Critical reflexivity would therefore support the development of a moral, social, political commitment, as required for the deployment of a more socially engaged professional practice.^42,43^ and for broader transformative social action.^4^

Finally, inspired by the work of Fook and Gardner,^45^ we also provide theoretical support for our understanding that the reflexive process does not take place in a vacuum. We thus identify reflexivity as a situated interactional process shaped by both individual, historical and structural factors, highlighting the link between social power dynamics and knowledge creation.

**Figure 1.**
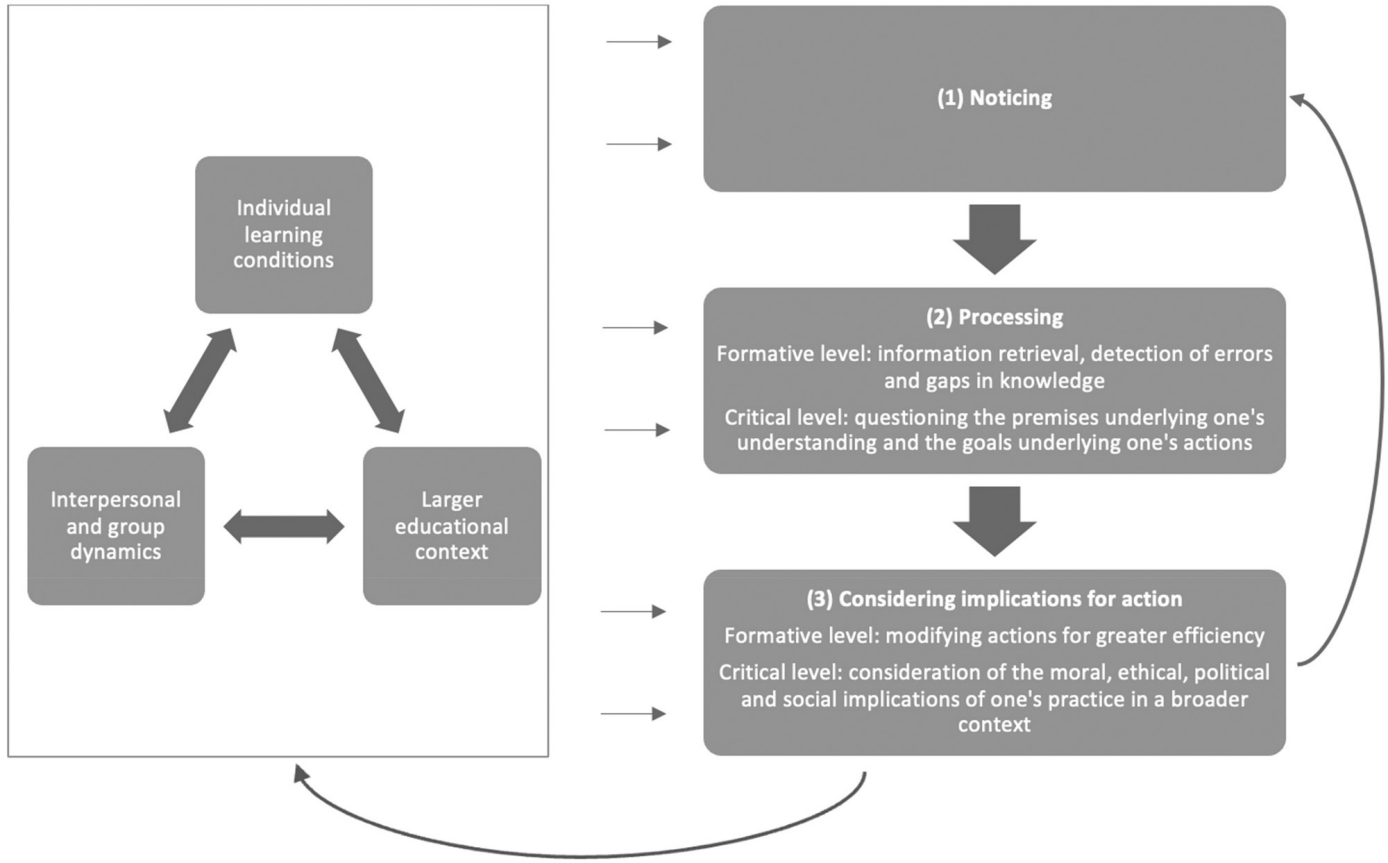
An integrative conceptual model for reflexivity through small group learning.

Figure 1 provides a schematic representation of an integrative model of the conceptual elements of reflexivity used in this study. It is based on the team learning model proposed by Dechant et al,^46^ which adopts a dynamic, open systems approach based on the premise that learning processes are influenced by factors (inputs) operating in several dimensions. As an addition to Dechant's proposal, we postulate that the different dimensions of inputs present complex interactions with one another. Adopting such a systemic view also requires us to think of a recursivity between reflexive learning outcomes and inputs, considering that the reflective process will have potential repercussions on individuals, groups and the larger context.

### Case selection strategy

Our case study focuses on the development of reflexivity among medical students who were involved in the discussion workshops of the compulsory MED-1210 course that hosted the intervention in Winter 2021. Our study thus targeted three groups among the eight interventional workshop groups. Each group was seen as a unique manifestation of the phenomenon under study, belonging to a larger set of potential cases.^29^ An in-depth study of these manifestations and their specificities, similarities and differences was thus expected to ultimately contribute to a better understanding of the phenomenon under study.^29^

In line with Stake's perspective,^29^ the criteria of relevance, diversity and learning potential guided the case selection process. Indeed, according to the author,^29^^(p4)^ rather than aiming for representativeness of the cases, the researcher should “carefully consider the uniqueness and context of the alternative selections, for these may aid or restrict our learnings.” Each group was thus chosen regarding the light it may shed on our research objectives, given its own particularities and originality. In concrete terms, selection was based on descriptive information informally collected for each group (participants' profiles, specific dynamics, etc.) as collated in a research logbook and on preliminary data explored by the first author (JM). The final selection was discussed with MCT until a consensus was reached.

### Data collection strategy

In case study research, it is widely recognized that data collection must take various complementary forms to achieve an understanding of the phenomenon under study in all its complexity.^47–49^ In this study, data collection was based on semi-structured individual interviews, non-participatory direct observation, secondary demographic data and the keeping of a research logbook. Table 2 presents a summary of the data sources for each of the three groups studied.

**Table 2.** Data sources for each of the cases.

	SEMI-STRUCTURED INDIVIDUAL INTERVIEWS	NON-PARTICIPANT DIRECT OBSERVATION	SECONDARY QUANTITATIVE DATA	RESEARCH LOG
Case 1	w/patients: *n* = 2 w/students: *n* = 4 w/phys.-instr.: *n* = 1	4 recordings/5 workshops	For all 12 student participants	Descriptive and reflective notes, apply to all groups
Case 2	w/patients: *n* = 2 w/students: *n* = 4 w/phys.-instr.: *n* = 1	n/a	For all 12 student participants	
Case 3	w/patients: *n* = 2 w/students: *n* = 4 w/phys.-instr.: n/a	5 recordings/5 workshops	For all 11 student participants	

#### Semi-structured individual interviews

The data collection strategy involved semi-structured interviews with patient-teachers, physician-instructors and students from each selected group. The only inclusion criteria considered were: (i) having participated in a selected workshop group and (ii) having consented to participate in a qualitative interview through the consent form that has been produced and distributed to all potential participants for the entire study (quantitative and qualitative components). All solicited patient-teachers and physician-instructors agreed to participate and were interviewed, except for one physician-instructor who did not respond to our invitation. Students were selected based on a convenience sampling strategy, on a *first come, first served* basis, until theoretical saturation of intra-group data was reached.

The interviews were all conducted by the first author between June 7 and July 20, 2021 and lasted between 30 and 100 min. Given the COVID-19 pandemic and associated restricted face-to-face contacts during our data collection period, interviews were all conducted online, *via* the Zoom videoconference platform.

As suggested by Creswell,^48^ the development of interview guides was largely based on the theoretical perspective adopted and our literature review. We wanted these guides to contribute to a flexible and fluid conversational interview process exploring predefined terms while having the possibility of engaging with emerging ideas identified as relevant and important by participants.^50^ The questions were organized into two sections exploring: (i) the participant's lived experience of the patient engagement intervention and (ii) the perceived reflexive effects and their production process. The preliminary guides were pre-tested and discussed with a MED-1210 course leader (for the instructors' guide), a patient-partner (for the patient-teachers' guide) and a student from the steering committee (for the students' guide). They were fine-tuned considering the feedback received to ensure their relevance to the research objectives, their responsiveness to the concerns of those involved, and to promote the fluidity and understandability of each question.

In preparation for the analysis, all interviews were digitally recorded and transcribed by the first author, with participants' consent.

#### Non-participatory direct observation

Data collection also involved, for the selected cases, non-participatory direct observation of the workshop sessions. Physician-instructors, who were responsible for the logistics of online meetings, were asked to record each of the meetings using the platform's recording function and to transmit it to the research team. The recording and transmission were subject to the ethical consent of all participants of a given group. As a result, observation data were available for only two of the selected groups. Also, subgroup discussions that took place in Zoom's break-out rooms were not recorded (as a limitation of the technological tool) and could not be observed.

An observation log was filled out by the first author as she reviewed the recordings. The log was used to take descriptive and reflective notes about the workshops' unfolding in relation to our research object and regarding certain specific questions raised during the analysis of interview data.

#### Secondary demographic data

To refine our description of each of the groups under study, descriptive demographic data generated as part of the quantitative component of the broader intervention research project was used to document the portrait of the students involved in the selected groups.

#### Research logbook

The first author kept a research logbook. This enabled her to maintain a reflexive posture and to keep in mind how her subjectivity and the uniqueness of her view of the world tint the entire research process as well as the conclusions drawn from it. Throughout the project, she also collected relevant descriptive and reflexive information concerning the practical, methodological, empirical or theoretical dimensions of the study. The log proved useful for tracing and clarifying the origins of certain interpretative avenues, which were exploited in formulating the study's results and conclusions.^51^

### Analysis

Our data analysis consisted of: (i) within-case analysis and (ii) cross-case synthesis.

#### Within-case analysis

The aim of this first stage of analysis was to understand the rich and singular story of each of the three workshop groups under study. With the research objectives in mind, we wanted to highlight the characteristics of these groups and understand their specific dynamics, with detail and nuance, to provide further context for the findings. Following Stake,^29^^(p123)^ this took the form of an extensive narrative description: “a body of relatively uncontestable data, not completely without interpretation, but a description not unlike they would make themselves had they been there.” From there, we performed a thematic analysis^52^ with the aim of identifying within-case patterns relevant to our research objectives, representing meaningful insights from each group regarding the reflexive effects of the intervention and their main processes of production. This analysis was carried out with our conceptual model of reflexivity in mind. At first, the first author reread the interview verbatims using the audio recordings to validate them. Once validation had been completed, the interviews were listened to carefully while reading the verbatim at the same time to become familiarized with the corpus. This was also an opportunity for the first author to take detailed notes on her impressions and ideas, thus highlighting certain emerging themes. At this stage, all interview transcripts, as well as relevant sections of the observation log and research logbook, were imported into NVivo for coding. We first sought to identify the smallest descriptive or thematic data segments that could be meaningfully interpreted in relation to the phenomenon under study, from which we drew, through an exercise in classification and grouping, some more general patterns. We gradually revised the coding grid and refined it until the identification of key within-case patterns. In line with Stake's proposal,^29^ through the entire analytical process, we paid attention to both replicative and single instance data, a process deemed significant for gaining a rich and detailed understanding of a group's particularities.

#### Cross-case synthesis

At this stage, we drew conclusions from the within-case patterns identified above regarding our research objectives by looking at the concordances and discordances noted between the themes that emerged from the within-case thematic analysis and by highlighting what was common and what was unusual about each of them within the larger educational context. We ensured constant dialectical back-and-forth between an individual case and the globality of the phenomenon under study, with a view to grasping its complex significance. This meant constantly going back and forth between raw data and evolving preliminary results to formulate new statements, hypotheses, and avenues of analysis. Those were either refined, nuanced or reinforced as our understanding of each case improved until satisfactory conclusions could be drawn regarding the research objectives.^28^

### Quality and rigor

The first author was the individual mainly responsible for the analytical process. Different strategies were implemented to ensure quality and rigor criteria were met.^48,53^ For instance, the preliminary results produced following a first within-case analysis were discussed in-depth by JM and MCT until a common view of the data and the coding grid was reached. Subsequently, JM regularly reported to MCT on the status of her analytical work and incorporated MCT's comments and suggestions to strengthen the description of emerging patterns and to gradually improve the coding strategy and coding grid. Peer validation was sought through a presentation and discussion of methodology and preliminary results with a team of researchers from outside the project. This allowed a more detailed and relevant analysis and reporting of the data. Two meetings were also held (one with a group of patients and then with the course leaders) to share preliminary results and welcome constructive feedback. This enabled verification, from multiple perspectives, of whether the preliminary results painted an accurate picture of the perceived reflexive effects of patient engagement in the MED-1210 workshops and of the main associated processes. It shed new light on the research and fostered deeper and richer analyses.^48,53^ Finally, the reporting of this study conforms to the Standards for Reporting Qualitative Research (checklist available as a supplementary file).^54^

## Results

### Descriptive groups' characteristics and dynamics

This section provides a rich description of the three groups under study to highlight the profile of the individuals who interacted within them and the power dynamics that unfolded in a common or differentiated way. This provides context for the following results. Table 3 summarizes the main descriptive features of the groups studied.

**Table 3.** Descriptive summary of the three groups under study.

	THE GOLDEN GROUP	THE ANCHORED GROUP	THE ENGAGED GROUP
Group operation modes	All deliberative discussions in full group	Deliberative discussions in 2 sub-groups; always the same sub-groups; return to plenary	Deliberative discussions in 2 sub-groups; patients alternately assigned to sub-groups; return to plenary
*Patients*			
Sex/Gender	Patient 1: W; Patient 2: M	Patient 3: W; Patient 4: W	Patient 5: W; Patient 6: M
Health situation	Patient 1: chronic condition with visible physical and cognitive manifestations; Patient 2: chronic condition with no visible manifestation; history of polytrauma	Patient 3: congenital malformation; mobility issues; history of chronic pain; Patient 4: cancer survivor; carer for a spouse with respiratory disabilities	Patient 5: chronic condition with mainly cosmetic visible manifestations; issues with access to care; Patient 6: mental health challenges; various risk factors and chronic conditions with no visible manifestations; history of chronic pain
Social and professional profile	Patient 1: pathways greatly impacted by the limitations imposed by health condition; Patient 2: community work and civic commitment; patient-partner in research and health bodies	Patient 3: community work and advocacy for the rights of disabled women; Patient 4: retired, worked for 25 years in human resources	Patient 5: living in poverty; advocate for the rights of people receiving social assistance; Patient 6: patient-partner in research and health bodies, peer helper; advocate for the rights of people with mental health issues
Participation approach/posture	Patient 1: sharing of an analysis of the clinical cases without explicit reference to one's own experience; Patient 2: sharing of real-life experiences, on a professional level; Both patients: occasional confusion in posture	Both patients: sharing of narratives deeply rooted in their own personal and sensitive life and health experiences; posture of the patient receiving care	Both patients: sharing of narratives rooted in lived experience but often oriented toward suggesting preferred clinical approaches and behaviors; socially committed posture referred to as not that of the “average patient.”
*Physician-instructor*			
Sex/Gender	M	W	W
Professional experience	Experienced family doctor	Experienced medical specialist	Medical resident
Teaching experience	30 years in undergraduate medicine programs	Long-standing experience in resident training; no teaching experience at the undergraduate level	N/A
Teaching stance	Incentive-based; kind, open and convivial; lack of proactivity in stimulating meaningful interactions	Incentive-based; directive behaviors (to control the content discussed and its framing)	Active; concerns about levelling out power imbalances
*Students*			
Sex/Gender	8 females and 4 males	8 females and 4 males	7 females and 4 males
Age	Most under 25 (*n* = 7)	Most under 25 (*n* = 9)	Most under 25 (*n* = 10)
Participation level to discussions	Low, participation limited to taking turns formulating answers to the instructor's questions	High, especially in sub-groups; viewed as respect for patients	High, especially in sub-groups; viewed as a learning opportunity to be seized
Posture	Professional	Professional ++	Professional

#### Case 1: the Golden Group (GG)

In our Golden Group, there were two patients, one physician-instructor and 12 medical students. We named it the *Golden Group* because, from the beginning, it was targeted as our benchmark. It was identified as such both for the especially sharp critical thinking of the two patients taking part, their high ability to speak up, and for the instructor's extensive experience both as a doctor and as an educator. Within the Golden Group, all the deliberative discussions took place in the full group, (ie: the students, patients, and instructor all together at all stages of the workshops).

##### The Golden Group's patients

Patient 1 was a woman suffering from a chronic illness with visible physical and cognitive manifestations affecting her speed of thought and reaction. Patient 2 was a man suffering from a chronic illness with no visible manifestations and with a history of polytrauma. Both patients were university graduates. Patient 1's academic and professional pathways have been greatly impacted by the limitations imposed by her health condition. The life and professional pathways of Patient 2 have been marked by community work and civic commitment. He acts as a patient-partner in several local and national research and healthcare bodies. Observation reveals an imposing man, both physically and intellectually, as supported by the words of Patient 1: “(…) the presence with a capital P of my partner. It was magnificent to see him go into his eloquence (…)” (Interview—GGPatient1).

Generally speaking, patients of the Golden Group were perceived by other participants as open to dialogue, non-judgemental, and engaged in the participative space. That said, Patients 1 and 2 had very different approaches to speaking up in the workshops. Patient 1 was more inclined to share an in-depth, sensitive, subjective analysis of the clinical cases without explicit reference to her own experience. Patient 2 was more likely to share real-life experiences to exemplify the points made, mainly based on past professional experiences.

Regarding the patients' participation posture, occasional confusion led both patients to look for solutions to the clinical cases with students rather than explicitly putting forward their specific perspective, as noted by a student: “They intervene as if they were students (…)” (Interview—GGStudent2).

##### The Golden Group's physician-instructor

The Golden Group's physician-instructor was a male, experienced family doctor. He has been teaching in undergraduate medicine programs for over 30 years, particularly in courses dealing with the psychosocial aspects of medical practice. In the workshops, he positioned himself as both an expert and resource person and adopted an animation style that encouraged students to express themselves through one-on-one instructor-student interaction. He thus tended to solicit student individual responses and opinions. One patient consistently mentioned that the instructor could have been more proactive in explaining the reason for patients' presence in the workshop and directive in stimulating patients-students' interactions. Similarly, some students outlined how the formal organization of discussions and speaking times has impacted students' possibilities to build on patients' specific knowledge and to engage in deeper and more meaningful discussions with them.

In the deployment of his role within the workshops, his attitude was kind, open and convivial. Students and patients alike found him interesting, nuanced, capable of “considering fundamental issues” and having an aptitude for transmitting his professional knowledge in a “not too technical” way.

##### The Golden Group's students

The Golden Group included eight female and four male students. Most were under 25 (*n* = 7) and came from privileged backgrounds. This group of students stands out for its low level of participation in discussions, which is consistent with the monitor's animation style. Some participating students also had previous experiences interacting with patients (eg, working in a pharmacy or rehabilitation clinic). Based on such experiences, some of them appeared to have developed a sense of self-efficacy in managing the doctor-patient relationship and thus considered patient participation in the workshops no longer had much to teach them: “I think I've already got used to talking to them [the patients], being exposed to them” (Interview—GGStudent2).

#### Case 2: the Anchored Group (AG)

In our second group, there were also two patients, one physician-instructor and 12 students. We called it the *Anchored Group* because of (i) the physician-instructor's posture, which tends to be more directive and task-oriented, and (ii) the patients' posture of participation, which is deeply rooted in lived experience. In this group, discussions took place in two subgroups with a return to plenary for key messages.

##### The Anchored Group's patients

Both patients were women. Patient 3 had a congenital malformation, severe mobility issues and a history of chronic pain. She had worked mainly in community organizations and was involved as an advocate for the rights of disabled women. Patient 4 was a cancer survivor and a carer for a spouse with respiratory disabilities. Both patients had a wide network of acquaintances, friends and family members who have experienced various life and health-challenging situations (eg, HIV infections, mental health issues, domestic violence).

Patients were perceived as open-minded, non-judgmental, and interested in students' perspectives. What stood out in particular was their generosity and sincere commitment to sharing real-life experiences, some of which were highly sensitive and personal, for the benefit of student learning.

##### The Anchored Group's physician-instructor

The Anchored Group's physician-instructor was a woman, experienced medical specialist. She had no experience of teaching at the undergraduate level but has long been involved in resident training. One patient shared her perception of a highly intelligent, rigorous, but intimidating person who exudes poise and authority: “she scared me a bit” (Interview—AGPatient3).

As for the Golden Group, this instructor seems to have positioned herself both as a content expert and as a resource person to guide the deliberative process, especially when in a full group. During the moments spent in sub-groups, some student and patient participants mentioned how the instructor moved from group to group and how this was perceived as a form of control and surveillance, with an impact on the flow of discussions: “I thought to myself: ‘she seems to be watching us’” (Interview—AGPatient4).

Such directive behaviors were also reflected in the adoption, during discussions, of strategies to maintain control over the shared contents and their framing. For instance, one patient mentioned the instructor's perceived tendency not to allow the group to engage in discussions on subjects she was less clinically familiar with (eg, HIV), despite the relevance and importance patients may attribute to them. Similarly, the instructor showed concern about maintaining interactions with patients in workshops within a professional framework (eg, the promotion of formal address), as a mean to teach medical professionalism: “I think it's important to send the message that this is what's expected of them [students] when they go into medical practice” (Interview—AGInstructor).

##### The Anchored Group's students

The Anchored Group included eight female and four male students. As for the Golden Group, most students were under 25 (*n* = 9) and came from privileged backgrounds. They were participative, receptive and good listeners. They did not hesitate to question patients directly, especially when in sub-groups where they felt they could express themselves without fear of being judged by an authority figure: “The fact that the teacher wasn't there all the time, I think that may have facilitated [subgroup] discussions” (Interview—AGStudent1). Students saw active participation in discussions as a mark of respect for patients: “When there's a patient, we have no choice but to be very assiduous” (Interview—AGStudent1). Consistently, they generally adopted a professional posture towards patients: “I think it [patient participation in the workshops] pushes us to be more professional and to put ourselves in the role of a doctor” (Interview—AGStudent1).

#### Case 3: the engaged group (EG)

In our third group, there were two patients, one physician-instructor and 11 students. We named it the *Engaged Group* for two key reasons: (i) the instructor's facilitation strategy, which was concerned with levelling out power imbalances and (ii) the participation of patients who were active advocates for equity in healthcare. Deliberative discussions took place in two subgroups, with a return to plenary for the key messages and conclusions.

##### The Engaged Group's patients

Patient 5 was a woman living in poverty, suffering from a chronic illness with a mainly cosmetic manifestation, and experiencing issues of equitable access to care. She was an advocate for the rights of people receiving social assistance. Patient 6 was a man suffering from mental health challenges and chronic illnesses with no visible manifestations. He also had a history of chronic pain. He was involved as a patient-partner in various bodies, as a mental health peer-helper, and as an advocate for the rights of people with mental health issues. Both patients had significant experience with speaking up about their life and health experiences.

For both patients, contributions to discussions, though rooted in lived experience, were often oriented toward suggesting preferred clinical approaches and behaviors to support equitable access and quality of care. Those contributions were globally driven by their socially committed stance, which seemed to disturb some students who did not see them as “average patients.”

Once again, it was mentioned how patients were open-minded, non-judgmental, and generous when it comes to sharing parts of their personal lives; however, the level of patient participation in discussions was perceived as not as high as in the other groups. Students highlighted how the low explicit correspondence between clinical cases and patients' own experience as well as the complexity of theoretical/biomedical aspects seemed to have limited patients' involvement in some discussions.

##### The Engaged Group's physician-instructor

The instructor involved in the Engaged Group was a woman, medical resident. She mainly positioned herself as a resource person. Even though she did address some formal content, students did not emphasize her role as an expert. She was perceived as a participant in the discussions and as a kind of a peer-helper: “She answered our questions and then knew how to relax us” (Interview—EGStudent4). This is consistent with her emphasis on levelling out power imbalances within the group and avoiding positioning herself as the sole holder of valuable knowledge. As a patient explained:

She said: 'First of all, we're all going to call each other by our first names, and then we're going to be on first-name terms for the whole gang. Because we're all equals. (…) She often repeated: ‘I'm not your boss. We're all here to learn’. (Interview—EGPatient6)

The instructor's posture was also reflected in the teaching environment favored during the workshops: in her bed after a night on call or in her living room, giving participants access to her personal living space. This set her apart from the Golden Group instructor who taught from his workspace, formally seated at his desk.

She was described as respectful, open and non-judgmental. Students spoke of her positive attitude and dynamism, which had an overall impact on group dynamics: “She brought beautiful colors to the group” (Interview—EGStudent1).

##### The Engaged Group's students

The Engaged Group included seven female and four male students. As for the other two groups, most students were under 25 (*n* = 10) and came from privileged backgrounds. They mentioned how they engaged in discussions in order to seize a precious learning opportunity: “We had to take advantage of the fact that they [the patients] were there” (Interview—EGStudent1). As for the Anchored Group, especially in break-out rooms, students questioned patients directly.

Most students maintained a professional/student-as-knower posture throughout the workshops, as illustrated by the words of one student for whom the professional imperative resulted in a limited level of participation in discussions: “I didn't mean to say anything wrong” (Interview—EGStudent3).

### Reflexive effects and their main processes of production

This section highlights the reflexive effects and their main processes of production derived from the within-case thematic analysis and their confrontation through cross-case analysis. The main themes refer to the stages of the reflexive process described in the second section: (i) noticing and processing and (ii) consideration of implications for action.

#### Noticing and processing

This section highlights reflexive effects and processes of the intervention at the two first stages of the reflective process. It unfolds in four sub-themes: (i) concretizing and anchoring otherwise disembodied content, (ii) bringing in other points of view, (iii) questioning one's convictions and beliefs about the self and the profession, and (iv) becoming aware of the social distance between the doctor and the patient.

##### Concretizing and anchoring otherwise disembodied content

Across all three cases, students emphasize how patient contributions to workshop discussions promoted the concretization of the theoretical content of the course (and, more broadly, of the undergraduate curriculum as a whole). Patient participation thus appears to have fostered student awakening to how medical theory applies in real life, leading to the consequent development of an extra layer of understanding giving meaning and anchorage to otherwise disembodied content, as illustrated by a student:

Let's say we saw an example [in a clinical case] and we thought, ‘well, maybe that's a bit far-fetched. (…) In the end, is it really going to happen?’ Our patient was like ‘yes, it happened to my daughter’. (Interview—AGStudent4)

Consistently, it was outlined how the participation of patients gave meaning back to the highly demanding undergraduate training pathway by relating it to its ultimate human and social goal, that is, caring for people:

We're no longer here just to learn, we're here because there are patients who will benefit from this learning. (Interview—EGStudent1)

According to students, such concretization has resulted in a greater interest in the course MED-1210, which they otherwise perceived as lacking practicality and addressing pointless issues. It also allowed students to concretely project themselves into their future professional practice, which acted as some kind of engaging trigger:

I like knowing that: ‘ok, this is really going to come in useful, I'm really going to have to make decisions like this, sort out ethical issues like this’. That's what turned me on. (Interview—AGStudent6)

##### Bringing in other points of view

Students from all three groups also highlighted how patients' contributions to discussions brought in other perspectives they would not have thought of otherwise since they naturally tend to apply to the clinical cases the professional/rational logic learned through their training:

As students, we looked at situations [ie, clinical cases] and then tried to think of laws, codes of ethics, all that … whereas patients went more with their common sense, their lived experience. (Interview—EGStudent5)

Such contact with other points of view was an opportunity to realize the limits of the sole clinical perspective in comprehending situations. It was mostly done by making visible the otherwise overlooked sensible narrative and non-clinical aspects surrounding clinical situations:

You really need to take the time to think about how you would feel (…). They [the patients], do it. We, read clinical cases. (Interview—GGStudent10)

This also allowed exposure of the plurality of perspectives applicable to clinical situations and the need to take them all into account to grasp all their complexity:

Now we [students] know that we can't necessarily know everything they [patients] think (…) We could see that our not simplistic but… obvious reflections aren't always applicable. (Interview—GGStudent10)

##### Questioning one's convictions and beliefs about the self and the profession

Data also enabled us to grasp how patient participation led some students to question their convictions and beliefs. This is illustrated within the Golden Group, where a student once perceived Patient 2's argument as a personal attack. Despite the anger and discredit felt at the onset, the situation led to the self-centered reflexive questioning of his own certainties and sense of self-efficacy in facing clinical situations, toward a humbler and more nuanced approach:

I kind of thought about it and then I said to myself, am I the problem in this story? (…) It reminded me that I'm not always right. (Interview—GGStudent2)

In this situation, the student was led to give an all-new value to patients' experiential knowledge: “What he [the patient] says is based on experience, so I have to take it into account” (Interview—GGStudent2).

Beyond such self-centered introspection, particularly in the Engaged Group, where patients explicitly shared their preferences regarding certain clinical behaviors and approaches, patients' contributions led to the critical questioning of medical professionalism as promoted in medical training. As one student put it:

What is considered professional? Sometimes, the line is a bit blurred. Here [patient 6] was saying how much he appreciated being familiar with his doctor. (…) But at the same time, we're told [in training] that we must stay very distant from the patient, not get close to him … stay professional. (Interview—EGStudent3)

In a similar vein, some students highlighted how patients' sensitive sharing of their lived experiences led them to grasp the omnipresence of emotions and values in the care relationship, thus promoting a renewed conception of such a relationship as fundamentally human and non-neutral: “As doctors, we're not robots, and patients aren't just diseases to be treated. On both sides, we're humans at heart” (Interview—EGStudent1).

Furthermore, for some Engaged Group students, the sharing of patients' harmful experiences of care has also led to some kind of indignation in realizing that what they conceive of as the basics of good medical practice are not embraced by all clinicians:

[Patient 5] told us that she was seeing [a specialist]. Just by the way he was, [the specialist] had already judged her, and this was interfering in the care process. (…) It's almost impossible for a doctor to do that! To hear that situation, it just made me go: ‘Come on!’ (Interview—EGStudent4)

##### Becoming aware of the social distance between the doctor and the patient

Patient participation also enabled some students, particularly in the Anchored and Engaged Groups where the students were more in contact with patients' personal stories, to become critically aware of the doctor's privileged social position and the distorted view of the world that may result from it: “Sometimes, I think our perceptions [as future doctors] is a bit altered by the [privileged] way we've always lived” (Interview—EGStudent3).

In the Anchored Group, where the students especially emphasized the poignancy of patients' shared experiences, this awareness enabled them to grasp with empathy the existence of other realities hitherto unknown to them. In such circumstances, patient participation also helped raise students' awareness of patients as human beings with potentially complex living contexts and recognition that appearances can sometimes be misleading:

It's crazy to see that a woman like that, who looked strong, who looked … who really gave off something good, had gone through all that. (Interview—AGStudent6)

Furthermore, in the Engaged and Anchored groups, where students were confronted with stories of stigmatization and exclusion, patient input has deconstructed prejudices against specific patient groups that might otherwise have been considered lost causes:

[Patient 6] had psychiatric problems. I find people with psychiatric problems intimidating. It's frightening. In society, these people don't have a good reputation, I think. Seeing him [patient 6] chatting with us, talking about his background, being super friendly, you realize: ‘We can really get through this!’ (Interview—EGStudent3)

#### Consideration of implications for action

This section highlights reflexive effects at the third stage of the reflexive process involving translating the lessons learned from the previous stages into actionable reflexive outcomes. This unfolds into two subthemes: (i) alternative ways of doing: concrete clinical behaviors; and (ii) alternative ways of being and thinking: core attitudes and values guiding medical practice.

##### Alternative ways of doing: concrete clinical behaviors

###### Involving patients in their own care

Among the clinical behaviors promoted by patient engagement is an approach aimed at actively involving patients in the planning of their own care in order to access other solutions and approach with flexibility the uncertainty surrounding singular complex clinical situations. As one student put it:

It's important to ask questions. Is it right for you and why? What would be better? What do you like about this option? What do you like less? All this must be included in the reflection. (Interview—AGStudent5)

###### Rethinking care trajectories

The acknowledgment of the plurality of life and health trajectories was also an occasion to realize that care needed to be tailored to patients' singular realities. For instance, students from the Anchored Group (where a patient discussed her mobility issues) reported new concerns about care coordination and logistics:

You're not necessarily going to move your patient all the time to get a little blood test, a little this, a little that. (…) You'll make sure it's all on the same day, not in two different hospitals. (Interview—AGStudent5)

Moreover, in a reflection highlighting doctors' limitations in addressing certain social issues and the crucial role of community organizations (as highlighted by some patients' narratives), several students stressed the benefits of turning to competent interdisciplinary and intersectoral resources to better meet patients' specific needs and expectations: “When I don't know too much about a subject, maybe I can (…) direct them to more appropriate resources than me” (Int—AGStudent6).

Some students also demonstrated a particular concern for continuity of care, seeing themselves, as future doctors, as taking on a patient continuous support role (rather than a referral role):

It's not just saying: ‘ah, go to your local [health center], there'll be a social worker there to help you’. It's about following them [the patients] along, being there for them. (Int—AGStudent5)

###### Communicating the right way

In all three cases, students emphasized how the presence of patients in the workshops led them to develop the ability to explain medical content in simple terms. They also highlighted how patients' contributions promoted their understanding of the importance of adapting interviewing techniques and ensuring that patients feel at ease within the doctor-patient relationship. Especially in the Golden and Anchored Groups, attention was drawn to the importance of having a vocabulary and attitude that are non-offensive to patients in recognition of the sensitive nature of certain topics:

We have to be careful of what we say. We have to choose our words. If we're talking about abortion, for example, we have to be sensitive in what we say. (Int—AGStudent1)

##### Alternative ways of being and thinking: core attitudes and values guiding medical practice

Students from all three groups highlighted how the reinvestment of lessons learned also translates to other ways of being and thinking as a doctor. They shared their desire to subscribe to models of practice rooted in values of openness, listening, humility, respect, and non-judgment. They also emphasize the crucial importance of empathetic practice, taking social distance into account:

That's not me anymore… not me living in my house with my family, studying medicine and living my life right now. [I have to shift my thinking to] if I were disabled, if I had all this experience, what would I want? (Interview—AGStudent6)

## Discussion

Through a qualitative case study design, this article aimed to shed light on the reflexive effects arising from a patient engagement intervention in an undergraduate medical course at UL as well as the main processes by which these effects are produced. It thus fills gaps in the body of knowledge concerning patient engagement in medical education by providing an in-depth and nuanced understanding of the *hows and whys* of patient engagement's effects on reflexivity in medical students.

In line with theories of reflexivity and as documented elsewhere,^55,56^ our results identify the meeting of perspectives at the origin of the reflexive process. By bringing patients', students' and instructors' points of view together in workshops, the intervention has created “a site for growth and transformation”^57^^(pS72)^ conducive to reflexivity among students. Even though we were dealing with students at the very beginning of their journey, as did Gross et al^56^—who were rather interested in patient engagement with interprofessional health students at the last year of their initial formation—, our study suggests that students' first stages of the reflexive process (noticing and processing) unfolded in some students on a critical level. Indeed, they called into question the epistemic foundations and professional requirements of the medical profession as well as its capacity to meet patients' needs and expectations. Our results also highlight students' raised awareness of the social distance between doctors' and patients' realities and, as suggested by Gross et al,^56^ promoted the deconstruction of prejudices, acknowledging that ways of thinking were based on questionable premises. Reflections were also expressed about the self and its openness to enter into dialogue with other challenging points of view, reminding that students can *a priori* be “reluctant to accept (…) a challenge to the assumed superiority of their ‘professional’ knowledge”^58^^(p318)^ and recalling the very objective of the dialogical approach: “the goal is not for everyone to agree, but for each perspective to evolve in some way.”^57^^(pS73)^ Interestingly, however, when it comes to the reinvestment of such reflection in action, students generally took a more formative approach, with a tendency to recite formal content rooted in the patient-centered approach. When asked about the applicability of reflective learning to practice, in a context where most undergraduate students had no clinical experience, they necessarily referred to some theoretically learnt contents on which they have intermediately built their ideal future model of practice. Such a finding highlights the limits of studying reflexivity in students holding no embodied and sensible experiential material to build on when it comes to creating new knowledge about one's professional practice. We suggest that future research looking at the effects of patient engagement on reflexivity in medical students take a more longitudinal perspective to see how effects evolve over time and how reflections translate into medical practice, beyond intention. Such a longitudinal perspective is all the more relevant as it will enable to consider, over the course of a student's journey, the mediating effects of elements from the curricula (formal, informal and hidden) that may impact the effects of patient engagement on reflexivity by fostering the reproduction of certain behaviors and thought patterns.^56,59,60^

As argued following our quasi-experimental study^22^ and in line with Dewey's definition of thinking as a process that always has reference to some context,^61^ our study also promotes the conception of reflexivity as a situated and contextual phenomenon.^31,45,62^ The results suggest that reflexivity unfolds in various ways, under the influence of a variety of multidimensional and interrelated individual and contextual considerations, to be better understood through the qualitative exploration of people's lived experiences.^2,4^ Within this study, the qualitative results derived from three groups whose singular stories were put into perspective with each other to shed light on reflexive processes also enabled us to uncover some conditions under which reflexive effects are more or less likely to unfold through patient engagement and to derive practical implications for medical schools. This highlighted the importance of:

### Reclaiming emotions as a primary source for productive and transformative learning

We first note that the type of narratives shared seem to have had an impact on the value placed by students on patients' knowledge and its consequent ability to contribute to students' engagement in a reflexive process. As observed in the Anchored and Engaged groups, value was placed on patients' sharing of real-life experiences of illness and the healthcare system, engaging students' emotions facing singular and often unfamiliar realities. Our study thus underlines the importance of emotions conveyed through narrative pedagogy in reflexive undergraduate learning and suggests that reflexivity unfolds beyond a cognitive and intellectual dimension.^62,63^ This calls into question the cartesian dualistic idea that has become a persistent *emotional rule* in medicine (and corollary in medical education) that, to be a good doctor, one must “evacuate emotion from reason.”^64^^(p.72)^ Following Boler,^65^ patient engagement in the context of the intervention turned out to be an opportunity for undergraduate students to reclaim their emotions as a legitimate part of their reflexive learning inquiry. Indeed, our results suggest emotions played a major role in shaping students' perceptions about patients' narratives and in the selection of what to pay attention to and what to explore further. This study thus contributes to the case for creatively engaging students in salient, meaningful, and emotionally charged learning experiences and then collectively acknowledging students' emotions as a primary source for productive and transformative reflexive learning.

### Acknowledging patient knowledge's breadth and richness

As documented in other studies^55,66,67^ and in line with the professional stance maintained by most students, it appears that patients were expected to participate from the perspective of the patient receiving care. This underlines the reductive way in which students tend to think about patients and their specific knowledge.^68^ Indeed, despite their acknowledged pedagogical value, patient contributions, when they are conceived of in terms of individual and subjective experiences and feelings, are often confined to a role of illustration for biomedical knowledge.^68,69^ Within our intervention, however, patients were not simply asked to testify about their individual experience of illness and care, but to actively participate in discussions. Such participation meant they had to mobilize their knowledge in all its breadth, richness and complexity, necessarily invoking tacit, cultural and embodied knowledge.^70,71^ Following Simon et al,^70^ in the context of the workshops, such patient knowledge guided the way patients approached the clinical cases, what they found important, what they wanted to discuss with students and how they did so. Whether or not they made explicit reference to lived experience of illness and care, such patient knowledge has the potential to reopen avenues of knowledge that have been neglected or not explored in depth.^68^ This calls for a renewed conception of the role of the patient-teacher in medical education, not only as the embodiment of theoretical/biomedical knowledge, nor as the bearer of other knowledge to be added to already plural learning, but as a means of moving learners towards an epistemological reflexivity concerning the scope of knowledge taught in the field.^68^

### Promoting transformative teaching and learning postures

Our results also shed light on how instructors, as the sole holders of formal power within the workshops, defined group norms, thus influencing patients' role and place, the valuation of patient specific knowledge and their ability to support reflexivity in students. Besides their behaviors and animation style, the instructors' formal power impacted how groups functioned (eg, a large group in the Golden group vs subgroup discussions in the Anchored and Engaged groups), which influenced the reflexive scope of deliberative exchanges. Indeed, as mentioned in other studies,^56,72–74^ our results suggest that conducting deliberative discussions in subgroups promoted proximity and risk-taking among participants, whereas large-group discussions were more formal and focused on questions formulated by the instructor. Subgroups have indeed proven to be a safe space for the sharing of more meaningful, sensitive, engaging, and emotionally arousing narratives and for asking questions. As highlighted in the group learning literature, we conceive such risky behaviors as conducive to the deployment of reflexivity since it allows the learner “to make, acknowledge, and reflect on mistakes and shortcomings, openly share information and perspectives, confront blind spots, consider different ideas, and experiment with unproven approaches.”^75^^(p1184)^ The instructors' position of power and its influence on the reflexive process unfolding among students emphasize the crucial importance of faculty training and support toward the deployment of a shared culture of patient engagement, promoting transformative teaching and learning postures.

#### Limits and avenues for future research

The results of this study should be interpreted while taking their limitations into account. In particular, students were selected for interviews based on a convenience sampling strategy, on a “first come, first served” basis. It is thus reasonable to think that those who raised their hand first were those who have had a positive experience with the workshops and were keen to talk about it. That said, students showed great enthusiasm for participating, and theoretical saturation was reached before all interested students were interviewed. We suspect students' participation was first boosted by their general interest in research. The involvement of the course leaders in the process of communicating with students about the project may also have had a positive impact.

Another limitation relates to online interviewing. Ethically speaking, no participant was excluded on the grounds of a lack of technological skills, a lack of access to software or an appropriate Internet connection, or discomfort with having their image transmitted via webcam.^76,77^ Participants were also well supported in their use of technology. Nonetheless, the data obtained may have been influenced by the fact of being online, a context where trust is more difficult to build between interviewer and interviewee.

The difficulty of accessing observational data is also a major limitation of this study. In one of the selected groups (and in many other groups not selected among cases), some students refused to allow the workshops to be recorded. Despite our ethical commitments, we suspect it was linked to students' lack of trust in the research process and its independence from the academic assessment process. In a context of high-performance, students did not want research materials in which they were likely to show their vulnerability to be at risk of being viewed by unwanted third parties. This underlines a particular issue related to the recording of learning activities in medicine for research purposes. Methodological research in medical education should address this issue to guide qualitative and mixed-method researchers in the best use of observation as a viable research strategy in the field.

## Conclusion

This case study was an opportunity to identify patient engagement in discussion workshops in an undergraduate medical course as a promising avenue to foster reflexivity in medical students and to understand the processes through which such reflexivity unfolds in such a context. It contributes important knowledge to the medical education literature, shedding new light on the value of patient engagement and its transformative potential in the field, as a catalyst for new ways of thinking and new perspectives for action. Our study also provides concrete action targets for medical schools wishing to commit to renewed educational postures and approaches acknowledging and formalizing patient active and meaningful engagement practices, rooted in the principles of epistemic justice, toward the development of reflexivity, conceived as a transformative way of being a doctor.

## Supplemental Material

sj-docx-1-mde-10.1177_23821205251324295 - Supplemental material for Fostering Reflexivity in Medical Students: Is Patient Engagement a Promising Avenue? A Qualitative Case Study Supplemental material, sj-docx-1-mde-10.1177_23821205251324295 for Fostering Reflexivity in Medical Students: Is Patient Engagement a Promising Avenue? A Qualitative Case Study by Julie Massé, Sarah Numainville and Marie-Claude Tremblay in Journal of Medical Education and Curricular Development
