# Supplementary material for: Fostering Reflexivity in Medical Students: Is Patient Engagement a Promising Avenue? A Qualitative Case Study
Source: J Med Educ Curric Dev. 2025 Mar 21;12:23821205251324295. doi: 10.1177/23821205251324295 (PMC11930470; doi:10.1177/23821205251324295)
Supplement: sj-docx-1-mde-10.1177_23821205251324295 - Supplemental material for Fostering Reflexivity in Medical Students: Is Patient Engagement a Promising Avenue? A Qualitative Case Study [file sj-docx-1-mde-10.1177_23821205251324295.docx]

Supplementary file – Standards for Reporting Qualitative Research (SRQR), checklist of items that should be included in reports of ***qualitative studies***

| No. | Topic | Reference in the text* |
| --- | --- | --- |
| Title and abstract | | |
| S1 | Title | p. 1 |
| S2 | Abstract | pp. 2,3 |
| Introduction | | |
| S3 | Problem formulation | Section 1, pp. 4,5 |
| S4 | Purpose or research question | Section 1, p. 6 |
| Methods | | |
| S5 | Qualitative approach and research paradigm | Sections 2.2 + 2.4 |
| S6 | Researcher characteristics and reflexivity | Section 2.3 |
| S7 | Context | Section 2.1 |
| S8 | Sampling strategy | Section 2.5 |
| S9 | Ethical issues pertaining to human subjects | p. 51 (declarations) |
| S10 | Data collection methods | Section 2.6 |
| S11 | Data collection instruments and technologies | Section 2.6 |
| S12 | Units of study | Section 2.5 |
| S13 | Data processing | Section 2.7 |
| S14 | Data analysis | Section 2.7 |
| S15 | Techniques to enhance trustworthiness | Section 2.8 |
| Results/findings | | |
| S16 | Synthesis and interpretation | Section 3.2 |
| S17 | Links to empirical data | Section 3.2 |
| Discussion | | |
| S18 | Integration with prior work, implications, transferability, and contributions to the field | Section 4, pp. 35-39 |
| S19 | Limitations | Section 4, pp. 39-40 |
| Other | | |
| S20 | Conflicts of interest | p. 52 |
| S21 | Funding | p. 52 |

* These references correspond to the sections and page numbers of the submitted manuscript.
